# An Unusual Foreign Body Causing Symptoms After 40 Years: A Case Report

**DOI:** 10.7759/cureus.91451

**Published:** 2025-09-02

**Authors:** Diana Marinha Cruz, José M. Seara, Daniel Ferreira Gonçalves

**Affiliations:** 1 Family Medicine, Local Health Unit of Alto Minho, Viana do Castelo, PRT; 2 Orthopaedics, Local Health Unit of Alto Minho, Viana do Castelo, PRT

**Keywords:** corrosion, foreign body, metallic object, metatarsal, residual foreign body

## Abstract

This is a clinical case of a 71-year-old woman who went to an urgent consultation for pain in the distal region of the sole of the right foot; she denied trauma or effort. On physical examination, there was an induration, which was painful on palpation. Ultrasound and X-ray evaluation showed a foreign body (FB) associated with a granuloma in the distal end of the metallic linear image, measuring 6.5 cm, projected at the base of the second metatarsal. When asked about the discovery, she remembered an episode of trauma with a rod from an umbrella, more than 40 years ago, which was supposedly removed by a healer. She was unaware that that piece of the object remained. Sent to traumatology, a partial extraction of the FB was performed. It showed extensive corrosion, causing a metallosis reaction. The patient kept a part of the FB with about 3 cm, with adequate healing, and she recovered completely.

## Introduction

The resilience and adaptability of the human body are often remarkable, particularly when challenged by unusual circumstances. In clinical practice, cases occasionally arise that defy expectations, highlighting the body’s impressive biological capacity to adapt in the face of adverse or unusual conditions. In certain situations, the body can tolerate foreign materials for prolonged periods without causing significant morbidity.

Eventually, retained foreign bodies (FBs), particularly metallic ones, may remain hidden in soft tissues for decades and are often only discovered following the delayed onset of symptoms [1] or as incidental findings in clinical imaging, as part of an evaluation of another problem [2]. Symptoms can emerge suddenly, even after long latency periods, due to repeated microtrauma, migration of the foreign object, or the development of local inflammatory or infectious processes [1].

According to literature, retained FBs are found in 7% to 15% of wounds in the emergency departments, and up to 38% are missed on initial physician evaluation [3]. Wood, glass, and metal accounted for 95% of the FBs seen [2,3]. FBs are a common clinical emergency that involves people of all ages, especially children under the age of five and older adults over 80 years old [4]. The incidence rate of FBs is higher in men than in women and higher among children, adolescents, and young adults [5]. Patients often wait weeks to months after initial injury to present for treatment [2,3]. The likelihood of persistent symptoms is low, and only 2% of retained FBs require removal later [6].

This report describes a rare case of a retained metallic FB in the foot, after a trauma, which remained asymptomatic for over four decades before manifesting clinical symptoms. This clinical case underscores not only the body’s tolerance to certain foreign materials but also the importance of thorough clinical history-taking and continued vigilance in cases of past penetrating trauma, even when previously deemed resolved.

## Case presentation

A 71-year-old female with no significant past medical history presented to an urgent consultation with a complaint of acute pain in the distal plantar aspect of her right foot, evolving over several days. She denied any recent trauma, overuse, or strenuous activity.

On physical examination, there was a well-defined, firm, and tender induration palpable in the distal plantar region. There were no signs of erythema, discharge, or systemic symptoms. Given the suspicion of plantar fascia pathology, soft tissue ultrasonography and plain radiography of the foot were requested.

**Figure 1:**
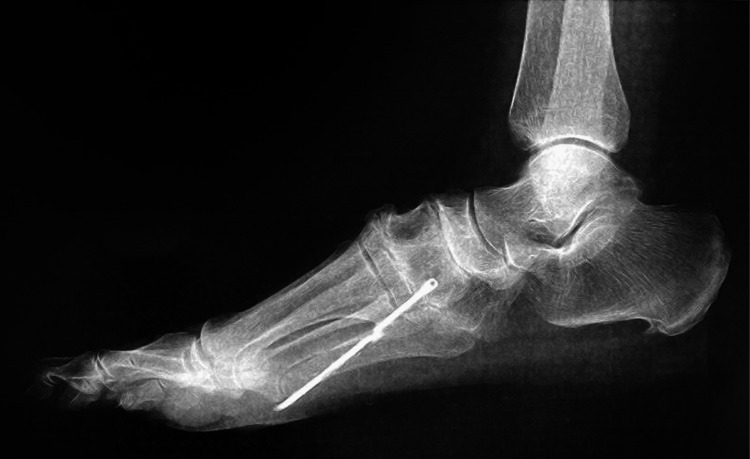
Diagnostic radiograph (lateral incidence). Foreign body projected at the base of the second metatarsal with linear metallic density, associated with small calcifications of a residual nature; plantar spur of the calcaneus; osteoarthritis of the middle foot and metatarsophalangeal and interphalangeal joint of the first toe.

**Figure 2:**
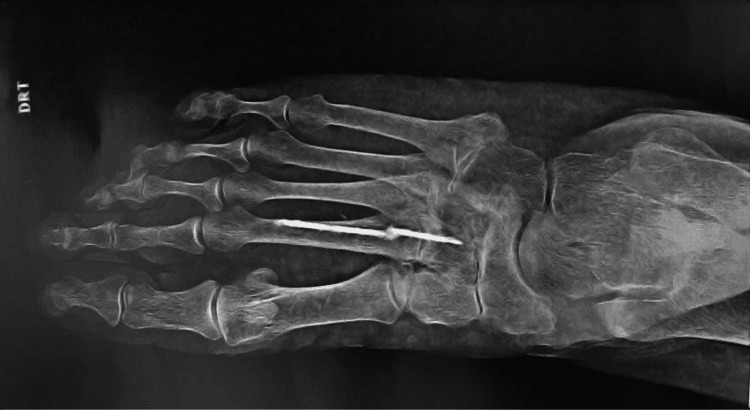
Diagnostic radiograph (anteroposterior incidence).

Ultrasonographic evaluation of the swelling revealed a FB granuloma associated with the distal end of a linear metallic, hyperechoic, oblique structure measuring 6.5 cm in length, situated at the base of the second metatarsal, surrounded by hypoechogenicity, suggestive of fibrotic changes; there were no associated fluid collections, no tendon alterations at the calcaneal insertion of the plantar fascia, and no associated inflammatory processes. The radiograph demonstrated an FB projected at the base of the second metatarsal with linear metallic density, associated with small residual calcifications; osteoarthritis of the midfoot and the first metatarsophalangeal and interphalangeal joints; and a calcaneal plantar spur (Figures 1, 2).

Upon further questioning, the patient recalled a penetrating injury with an umbrella rod more than 40 years earlier. At that time, the object was removed by a traditional healer; however, she neither sought medical evaluation nor underwent diagnostic imaging, and was therefore unaware of the persistence of any retained foreign material.

The patient was referred to the orthopedics department, where the risks and benefits of surgical removal were explained. She agreed to the procedure and signed the free and informed consent.

**Figure 3:**
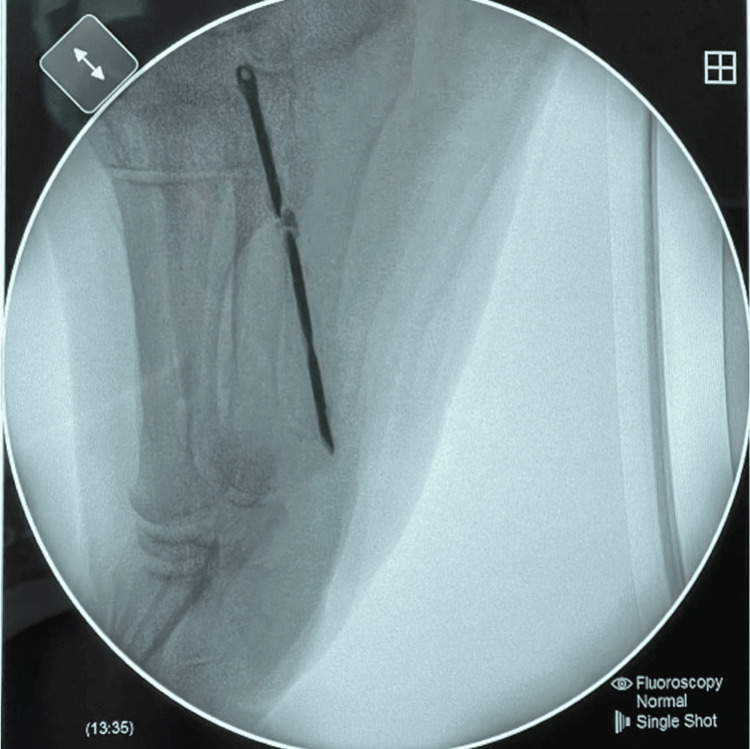
X-ray taken during the operation.

The surgical exploration was performed in the operating room under combined anesthesia, with the patient in the supine position, a pneumatic tourniquet applied to the right lower limb, and identification of the FB with a fluoroscope (Figure 3).

**Figure 4:**
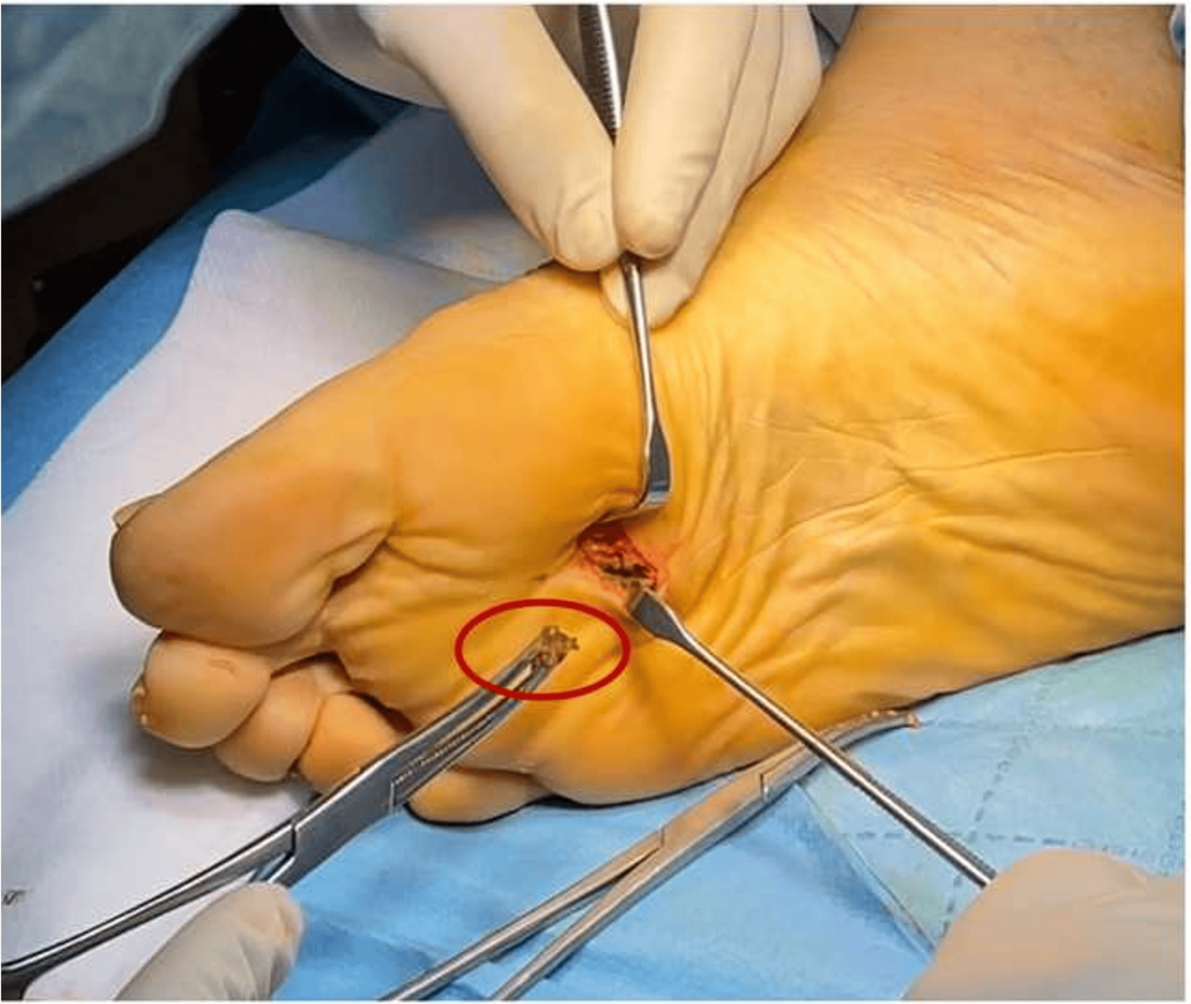
Metallosis from the surrounding soft tissues.

**Figure 5:**
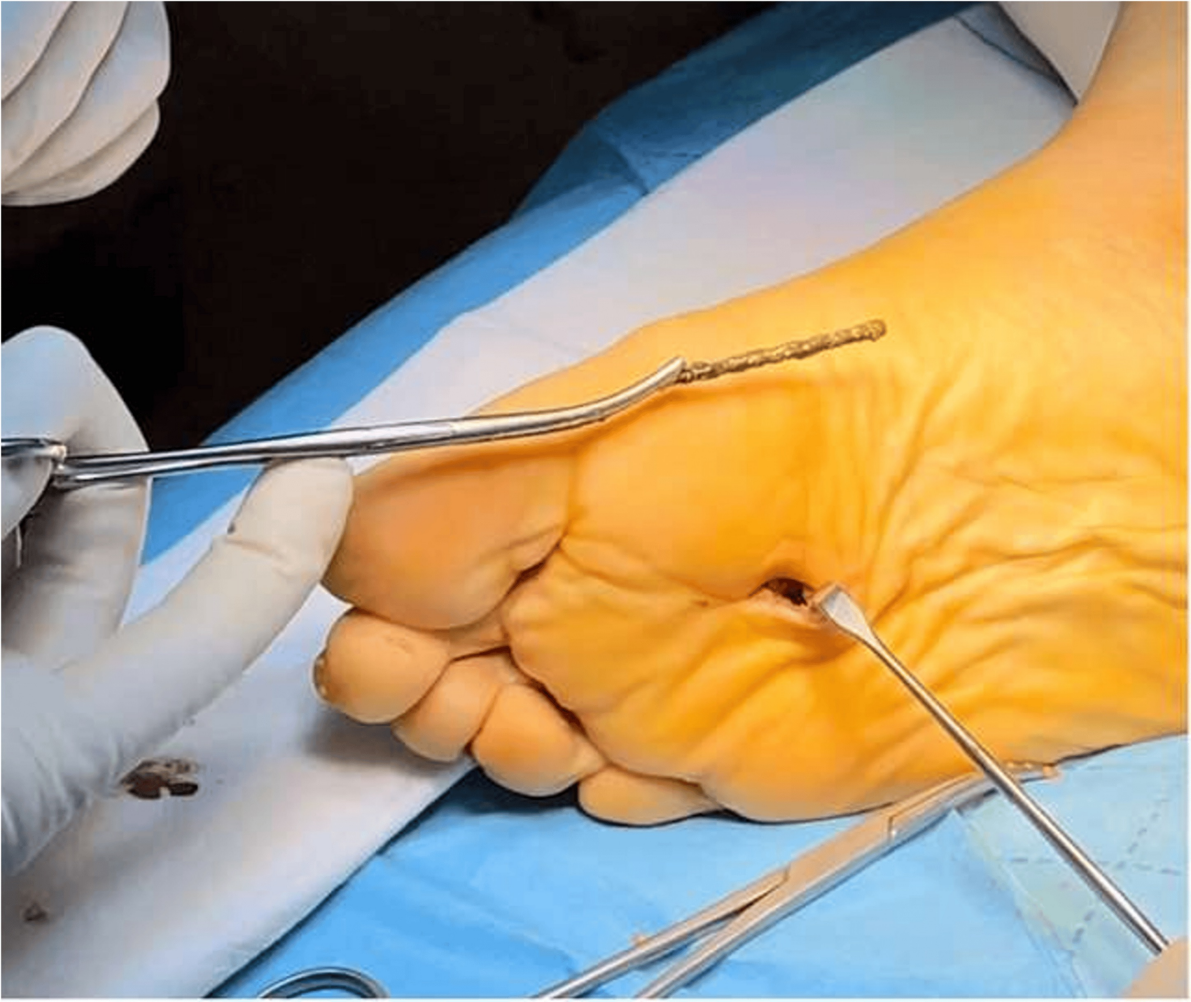
Extraction of the metallic object.

**Figure 6:**
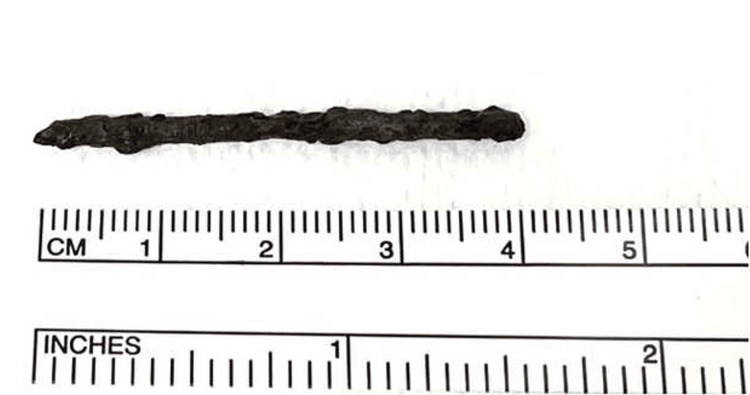
Size of the foreign body (umbrella rod) removed.

The FB was extracted through a plantar incision and was found to be extensively corroded, causing a metallosis reaction in the surrounding tissues (Figure 4). However, the extraction of the FB was only partial, as it had been sectioned into two parts (Figures 5, 6).

**Figure 7:**
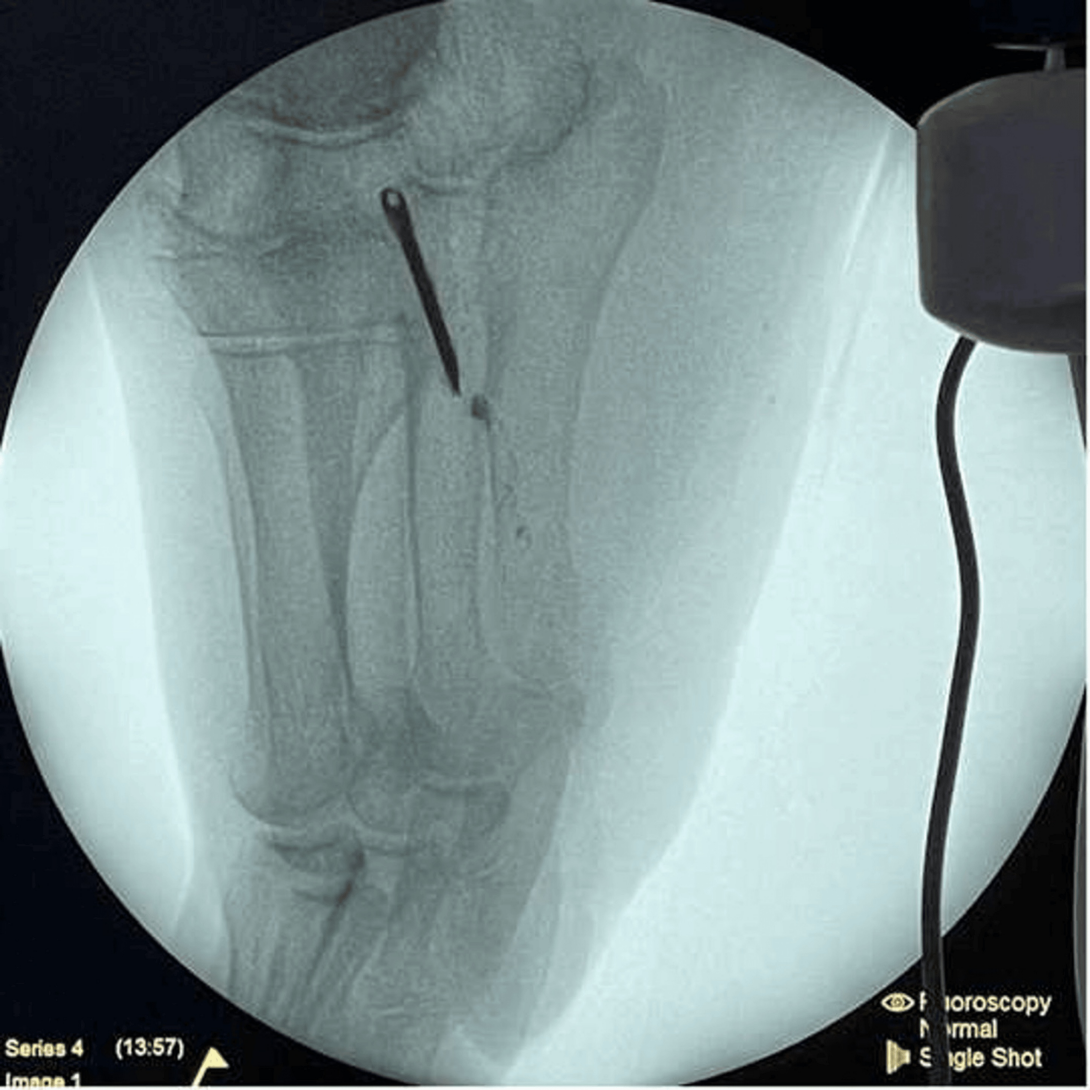
Final radiograph with the residual foreign body.

Attempted removal of the proximal portion through a second incision near the tarsometatarsal joint of the second ray [7] was unsuccessful, as its location in the metatarsal did not allow for safe removal without risking damage to the surrounding structures. Standard analgesia and antibiotic prophylaxis protocols were followed, and the patient's tetanus immunization history was updated. The patient was instructed to offload the operated limb for two weeks, followed by progressive weight-bearing as tolerated. She was informed of the presence of a retained FB fragment, approximately 3 cm in length, in the metatarsal region (Figure 7), and was counseled regarding potential complications, including discomfort, intermittent or trauma‑related pain, infection, chronic inflammation, and functional impairment. Management options were discussed, encompassing a new attempt at surgical removal versus conservative monitoring. The patient was advised on the importance of appropriate medical follow‑up to promptly identify and manage any arising issues.

Subsequently, the postoperative course was uneventful, with appropriate wound healing and complete resolution of symptoms. She made a full recovery and was able to walk up to 10 kilometers without complications during the two years of follow-up.

## Discussion

This is a rare case of a 71-year-old woman with a metallic FB retained in the foot for an extended period after the trauma, without symptoms. Despite surgical removal, a residual fragment remained; nevertheless, the patient achieved full recovery without apparent complications.

Although puncture wounds are common injuries, retained FBs after those incidents are rare [8], especially after proper exploration and management of the wounds [9,10]. In addition, patients may not express concern for FBs after an injury [9]. Undetected material can result in complications, including delayed wound healing, infection, and tissue damage, but the severity of these complications varies widely [1,3], according to the type of material and the patient’s immune status [11].

The foot is the second most common location of FBs, with the hand being the most frequent [12,13], but they are still uncommon in clinical practice, especially when remaining asymptomatic for long periods. According to the literature, the FBs are usually removed anywhere from the day of injury to 20 years later [2]. This is a rare case of four decades of delay in detection and symptoms. Moreover, one may wonder how, 40 years ago, it was possible to recover from this injury without adequate medical care and stay four decades without symptoms or complications.

Long-term retained FBs may remain silent until changes in material integrity or host immune response trigger clinical manifestations. Over prolonged periods, material degradation such as corrosion or chemical leaching can provoke localized tissue irritation and chronic low-grade inflammation. This inflammatory milieu may remain subclinical until disrupted by mechanical trauma, overuse, or changes in the host immune response, leading to symptom manifestation [1,2]. We question whether the reason why it has only now caused symptoms could be due to the fact that the object split into two parts spontaneously, or if it was for some other reason, which one we cannot clearly identify.

The development of symptoms decades after the initial injury is rare and may also be triggered by local tissue reactions such as granuloma formation or metallosis, and may even cause pseudotumors [13,14]. In this case, there was metallosis surrounding the FB, which is defined as the presence of free metal particles in the tissue - a rare phenomenon seen in orthopedic practice [15]. There is a pathological response to metal debris, typically arising from the wear, corrosion, or oxidation of metallic implants - well described in the context of metal-on-metal (MoM) joint arthroplasty - or from retained fragments [15,16]. Metallosis results as a syndrome of metal-induced synovitis, manifesting as abnormal dark macroscopic staining of soft tissues, and can yield local pain and inflammation as severe local reactions such as chronic synovitis and soft tissue necrosis, as well as systemic toxicity [15-18]. It can also induce systemic effects via an immunological response to circulating metal debris in the blood and lymphatic system, manifesting as constitutional symptoms such as nausea, vomiting, malaise, cognitive impairment, hematological abnormalities, cardiomyopathy, and neuromuscular changes [15-18]. Fortunately, this patient exhibited no systemic manifestations; however, vigilance is warranted as such manifestations may arise in different clinical contexts and in other patients.

This case highlights the importance of thorough clinical history-taking and consideration of remote traumatic events in the differential diagnosis of unexplained pain in the foot or other anatomical regions in elderly patients. However, it should be noted that unusual FBs can also appear in other age groups, particularly in patients of other nationalities, which is increasingly common with the emigration of patients whose countries have very different social, cultural, and health conditions [19]. Imaging modalities, including ultrasonography and radiography, are essential for the detection and characterization of FBs [20].

Surgical management should be tailored to the individual case, balancing the risks of extraction against the potential for ongoing tissue reaction. Removal can be difficult and time-consuming, and the potential damage to tissues caused by the procedure must be weighed against the risk posed by a particular FB. Metallic FBs may be removed without difficulty if superficial and distant from tendons, nerves, or blood vessels [8], which was not the case here. When removal might cause damage, metallic FBs can be left in place unless symptoms occur or infection is present [1,8]. In this case, the orthopedic surgeons decided to abandon the extraction of the proximal portion of the FB after an unsuccessful attempt, as its location did not allow for safe removal without risking damage to the surrounding structures.

## Conclusions

This case underscores the extraordinary capacity of the human body to tolerate retained foreign materials for prolonged periods. Clinicians should maintain some suspicion for FBs in patients presenting with atypical localized pain, even when the inciting event may have occurred decades earlier.

Although a fragment of an FB remained on her foot, this did not prevent the patient from fully recovering and continuing to walk, without complaints, during two years of follow-up. However, it is unpredictable if any other symptoms may develop in the future. In medicine, unusual cases, although infrequent, lead us to conclude that anything can happen, without it being possible to predict both the diagnosis and the results.
